# DNA damage-induced histone H1 ubiquitylation is mediated by HUWE1 and stimulates the RNF8-RNF168 pathway

**DOI:** 10.1038/s41598-017-15194-y

**Published:** 2017-11-10

**Authors:** I. K. Mandemaker, L. van Cuijk, R. C. Janssens, H. Lans, K. Bezstarosti, J. H. Hoeijmakers, J. A. Demmers, W. Vermeulen, J. A. Marteijn

**Affiliations:** 1 Department of Molecular Genetics, Oncode Institute, Cancer Genomics Netherlands, Erasmus Medical Centre, Rotterdam, The Netherlands; 2 Department of Proteomics, Erasmus Medical Centre, Rotterdam, The Netherlands

## Abstract

The DNA damage response (DDR), comprising distinct repair and signalling pathways, safeguards genomic integrity. Protein ubiquitylation is an important regulatory mechanism of the DDR. To study its role in the UV-induced DDR, we characterized changes in protein ubiquitylation following DNA damage using quantitative di-Gly proteomics. Interestingly, we identified multiple sites of histone H1 that are ubiquitylated upon UV-damage. We show that UV-dependent histone H1 ubiquitylation at multiple lysines is mediated by the E3-ligase HUWE1. Recently, it was shown that poly-ubiquitylated histone H1 is an important signalling intermediate in the double strand break response. This poly-ubiquitylation is dependent on RNF8 and Ubc13 which extend pre-existing ubiquitin modifications to K63-linked chains. Here we demonstrate that HUWE1 depleted cells showed reduced recruitment of RNF168 and 53BP1 to sites of DNA damage, two factors downstream of RNF8 mediated histone H1 poly-ubiquitylation, while recruitment of MDC1, which act upstream of histone H1 ubiquitylation, was not affected. Our data show that histone H1 is a prominent target for ubiquitylation after UV-induced DNA damage. Our data are in line with a model in which HUWE1 primes histone H1 with ubiquitin to allow ubiquitin chain elongation by RNF8, thereby stimulating the RNF8-RNF168 mediated DDR.

## Introduction

DNA integrity is constantly threatened by endogenous and exogenous DNA damaging agents. DNA damage interferes with transcription and replication, causing mutations, chromosomal aberrations and cell death, which may eventually induce malignant transformation and aging^1^. To counteract these deleterious effects cells have evolved an intricate network called the DNA damage response (DDR). This consists of signalling pathways that regulate cell cycle checkpoints and apoptosis, and a set of highly specialized DNA repair mechanisms each capable of repairing a specific subset of DNA lesions. UV-induced helix distorting lesions are typically repaired by nucleotide excision repair (NER)^2^. Helix distorting lesions located at any position in the genome are recognized by the global genome repair (GG-NER) proteins XPC and the UV-DDB complex. DNA lesions in the transcribed strand of active genes that cause stalling of RNA polymerase II initiate transcription-coupled repair (TC-NER). After damage recognition the DNA helix surrounding the lesion is unwound by TFIIH, which together with XPA verifies the lesion^3–6^. Next, RPA stabilizes the repair complex and positions the endonucleases XPG and ERCC1/XPF to excise the damaged DNA^7^. Before the single stranded DNA gap is filled by DNA synthesis by PCNA and the DNA polymerases δ, ε or κ^8^ it activates ATR signalling, which subsequently results in phosphorylation of histone H2AX on serine 139 (yH2AX)^9–11^. Phosphorylation of H2AX is a major DDR signalling event initiating the recruitment of many DDR-factors to activate cell cycle checkpoints and stimulate repair. This is induced by many types of genomic insults, such as DNA double strand breaks (DSBs) and stretches of ssDNA following replication fork stalling or NER-mediated excision^12,13^. MDC1 is directly recruited to yH2AX and functions as a scaffold protein crucial for the recruitment of many downstream DDR factors, such as RNF8, 53BP1 and BRCA1^10,14,15^.

The UV-induced DDR (UV-DDR) likely controls the successive reaction steps of NER and signalling pathways and regulates proper functioning of the involved proteins. A growing number of different post translational modifications (PTMs) have been reported in response to UV exposure, including phosphorylation^16^, ubiquitylation^17,18^, SUMOylation^19–21^ and PARylation^22,23^, likely to allow swift and reversible regulation of the UV-DDR. For example, the UV-damage-specific CRL4^DDB2^ E3-ligase complex, containing DDB1, DDB2, Cul4 and RBX1, ubiquitylates the GG-NER damage recognition factors DDB2 and XPC^24^. However, not only NER factors are regulated by ubiquitylation, also histones H2A^25,26^, H3 and H4^27^ are ubiquitylated in response to UV, events dependent on the CRL4^DDB2^ complex and other NER factors. This ubiquitylation of histones results in destabilization of nucleosomes thereby stimulating NER^26^. In addition, histone H2A is ubiquitylated in the RNF8-mediated DDR signalling pathway leading to the recruitment of downstream factors, such as 53BP1 and BRCA1^10^. In contrast, UV damage-induced stalling of RNA polymerase 2 triggers Ubp8 and Ubp10-dependent deubiquitylation of histone H2B, which is suggested to stimulate repair of lesions in actively transcribed genes^28^.

To identify UV-induced changes in protein ubiquitylation in an unbiased manner, we performed an enrichment procedure for ubiquitylated peptides^29^ in combination with quantitative proteomics. Tryptic digestion of ubiquitylated proteins results in specific peptides containing a Lys-ϵ-Gly-Gly (di-Gly) remnant, i.e. the two C-terminal ubiquitin glycine residues covalently attached to the ε-amino group of ubiquitin modified lysines. Ubiquitylated peptides can be enriched by immunopurification using antibodies that specifically recognize this di-Gly remnant, which will increase the identification efficiency of ubiquitylation sites on proteins by MS^29–31^. Using this approach we identified histone H1 as one of the most UV-induced ubiquitylated proteins, with multiple UV-induced ubiquitylation sites. A significant part of these histone H1 ubiquitylation events is mediated by the E3 ligase HUWE1. Recently, also Ubc13 and RNF8 have been shown to ubiquitylate histone H1 at DSBs leading to the recruitment of RNF168 and 53BP1^32^. RNF8 generates K63 linked poly-ubiquitin chains on H1, most likely on pre-existing ubiquitylation sites^32^. Based on this, we propose a model in which HUWE1 primes histone H1 by ubiquitylation to provide substrates on which RNF8 can generate poly-ubiquitin chains. In line with this model, depletion of HUWE1 results in a reduced damage signalling, as shown by the decreased recruitment of RNF168 and 53BP1 to sites of DNA-damage, while MDC1 and RNF8 remained unaffected, indicating that HUWE1 affects the RNF8-RNF168 pathway downstream of RNF8.

## Results

### Histone H1 is a major target of UV-induced ubiquitylation

To identify differentially ubiquitylated proteins in response to UV-induced DNA damage we isolated di-Gly modified peptides from mock treated, light (K0R0) labelled U2OS cells and from UV-irradiated (16 J/m^2^), heavy (K6R10) labelled U2OS cells using an anti-Lys-ϵ-Gly-Gly antibody (Supplemental Fig. S1a). Di-Gly enriched peptide mixtures were analysed by LC-MS/MS. Immunoprecipitation with the di-Gly antibody enriched the amount of identified ubiquitylated peptides to 35% of the total amount of identified peptides (Supplemental Table S1).

Three independent replicate experiments were performed and peptides were identified and quantified using MaxQuant software^33^. Within these experiments 5467 specific ubiquitylation sites in 3393 proteins were quantified. In total 250 di-Gly-modified peptides showed an increased abundance (UV/mock Log_2_ SILAC ratio > 0.75) in response to UV and 179 di-Gly peptides were found less (UV/mock Log_2_ SILAC ratio < −0.75) after UV (Supplemental Fig. S1b,c and Supplemental Table S1). We identified several peptides from proteins that were previously described to be ubiquitylated in response to DNA damage, including XPC^19,24^, DDB2^34^, and FANCD2^35^ (Supplemental Fig. S1c), providing the proof of principle of this approach. While analysis of di-Gly modified peptides cannot reveal whether a protein is modified with a mono-ubiquitin or with a linkage-specific ubiquitin chain, the relative abundance of site-specific di-Gly modified ubiquitin peptides, which are indicative for the types of ubiquitin chains formed, can be quantified. In line with previously reported data^36,37^, we observed a 1.5 fold UV-induced increase in di-Gly modified ubiquitin peptides at lysine 6 (K6), while the abundance of all other di-Gly modified ubiquitin peptides remained largely unaffected (Supplemental Fig. S1d). This suggests that the overall amount of endogenous K6-linked ubiquitin chains is increased after UV-induced DNA damage, indicative for a role of this atypical ubiquitin chain^38^ in the UV-DDR. Altogether these data show the validity of our approach to isolate, identify and quantify UV-induced ubiquitylated peptides.

**Figure 1:**
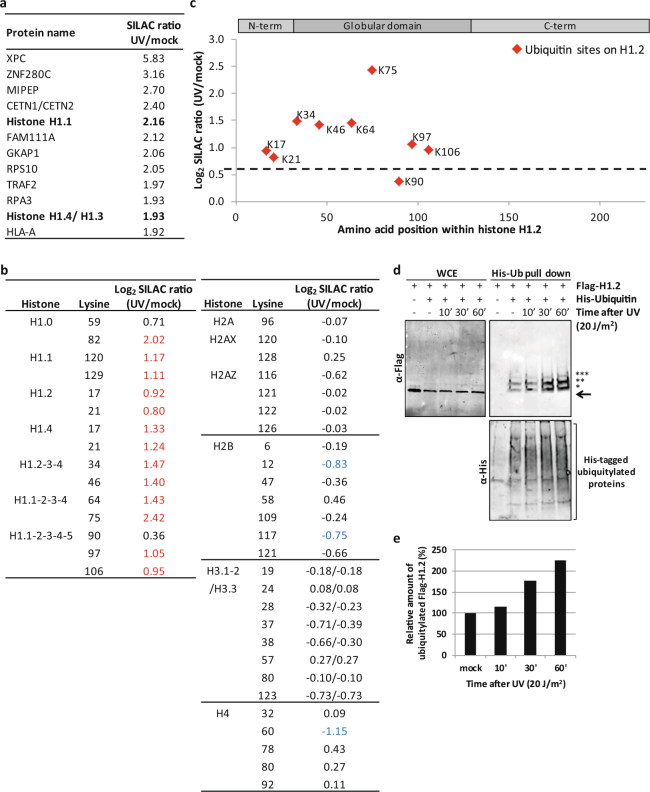
Histone H1 is ubiquitylated in response to UV. **(a)** List of the most UV-responsive proteins identified in the di-Gly IPs combined with mass spectrometry in U2OS cells. UV/Mock SILAC ratio is depicted, as determined by Maxquant analysis based on all peptides derived from di-Gly immunopurifications from 3 independent experiments. **(b)** List of all quantified ubiquitylation sites on histones. Log_2_ UV/Mock SILAC ratios as quantified by Maxquant analysis from 3 independent experiments are depicted. More abundant (Log_2_ > 0.75) ubiquitylation sites following UV exposure are shown in red and less abundant (Log2 < −0.75) ubiquitylation sites in blue. When di-Gly modified peptides could not be assigned to specific histone variants due to sequence similarities, all possible variants to which these peptides could be addressed are listed. **(c)** Graphical representation of the log_2_ UV/mock SILAC ratios for the quantified histone H1.2 ubiquitylation sites plotted against the respective lysine positions within histone H1.2. Quantification by Maxquant analysis from 3 independent experiments. Histone H1 domains are plotted above. Almost all identified ubiquitylation sites are more ubiquitylated in response UV and located within the globular domain of Histone H1.2. **(d)** U2OS cells transfected with His-Ubiquitin and FLAG-H1.2 were UV-C irradiated at the indicted times before cell lysis. Isolated His-tagged ubiquitylated proteins were analysed by immunoblotting using anti-His and anti-FLAG antibodies as indicated. WCE: whole cell lysate. The arrow (←) indicates the unmodified form of FLAG-H1.2. The asterisks (*) indicate ubiquitin-modified forms of FLAG-H1.2 and the amount of asterisks indicates the expected number of conjugated ubiquitin molecules based on the shift in mass of histone H1. Cropped westernblots are shown, full blots can be found in supplemental information. **(e)** Quantification of ubiquitylated FLAG-H1.2 signals on western blot after His-Ubiquitin enrichment. Data was normalized to the mock treated signal.

To determine which biological pathways are regulated by ubiquitin in response to UV-irradiation, proteins containing UV-induced ubiquitylation sites were subjected to gene ontology (GO) enrichment analysis. As expected, the functional protein network with the GO-term ‘cellular response to DNA damage stimulus’ was enriched, as represented by several DNA repair proteins. In addition the GO-term ‘chromosome organization’ was enriched (Supplemental Fig. S1e). Interestingly, this biological pathway is mainly represented by several variants of the linker histone H1. Histone H1 functions in chromatin compaction by binding to the nucleosome near the DNA entry and exit point^39^. Multiple histone H1 variants are among the proteins that were identified with the highest fold increase in ubiquitylation in response to UV (Fig. 1a). As the globular domain of histone H1 is highly conserved, it is difficult to distinguish for each identified peptide from which H1 variant it originates. However, we do find variant-specific peptides that are more ubiquitylated upon UV-damage for histone H1.0, H1.1, H1.2 and H1.4, indicating that UV-induced ubiquitylation is probably occurring on most histone H1 variants (Fig. 1b). Histone H1 peptides not modified by ubiquitin do not change in response to UV (UV/mock log_2_ SILAC ratio between 0.75 and −0.75) (Supplemental Table S1), indicating that the increase in di-Gly modified histone H1 peptides is caused by increased ubiquitylation, rather than increased histone H1 expression levels. In contrast to histone H1, the SILAC ratios of identified ubiquitylated peptides derived from core histones were barely changed after UV-damage (Fig. 1b). However, we do find a loss of H4K60Ub and several histone H2B sites to have reduced ubiquitylation levels after UV irradiation, in line with the described H2B deubiquitylation after RNA polymerase 2 stalling in yeast^28^ (Fig. 1b). Of note, previous described damage-induced core histone ubiquitylation sites^25–27,40,41^ were not identified. This might be caused by the very short peptides resulting from tryptic digestion of the lysine and arginine rich histones, which cannot be identified by MS. Interestingly, while thus far most DDR-associated ubiquitylation events on core histones are site-specific, e.g. H2A ubiquitylation on K13/15^41^ and K119^26^, we identified multiple ubiquitylation sites within histone H1, of which almost all are highly UV-responsive, suggesting that histone H1 ubiquitylation is less site-specific (Fig. 1b,c). our data show that histone H1 is one of the major targets for ubiquitylation after UV-damage and is the most ubiquitin regulated histone protein.

To validate the UV-induced histone H1 ubiquitylation, as detected by quantitative MS, FLAG-tagged histone H1.2 (FLAG-H1.2) and 6xHis-tagged ubiquitin (His-Ub) were transiently expressed in U2OS cells. Transfected cells were mock treated or UV-irradiated (20 J/m^2^) and lysed at the indicated time points (Fig. 1d) in a denaturing buffer to disrupt protein-protein interactions and inactivate the 26 S proteasome and deubiquitylating enzymes. His-tagged ubiquitylated proteins were isolated and equal pulldown efficiency was confirmed by western blot (Fig. 1d, lower panel). Next, the ubiquitylation status of histone H1.2 following UV damage was addressed using anti-FLAG staining. Interestingly, three FLAG-H1.2 bands, differing approximately 8 kDa from each other in size, were identified in the non-irradiated sample (Fig. 1d, upper right panel). As they specifically co-purify with His-tagged ubiquitin, these larger bands most likely represent histone H1 species modified with 1, 2 or 3 ubiquitin entities. Since multiple histone H1 ubiquitylation sites were identified by MS (Fig. 1b,c), these different ubiquitylated forms might correspond to histone H1 modified with mono-ubiquitin on 1–3 different lysine residues, although H1 poly-ubiquitylation of relative short ubiquitin chains cannot be excluded. The amount of FLAG-H1.2 co-purifying with His-Ub is increased 30 min and 1 h after UV irradiation (Fig. 1d,e), confirming that histone H1 ubiquitylation is indeed increased in response to UV-irradiation.

### The UV-induced histone H1 ubiquitylation is dependent on the E3-ligase HUWE1

**Figure 2:**
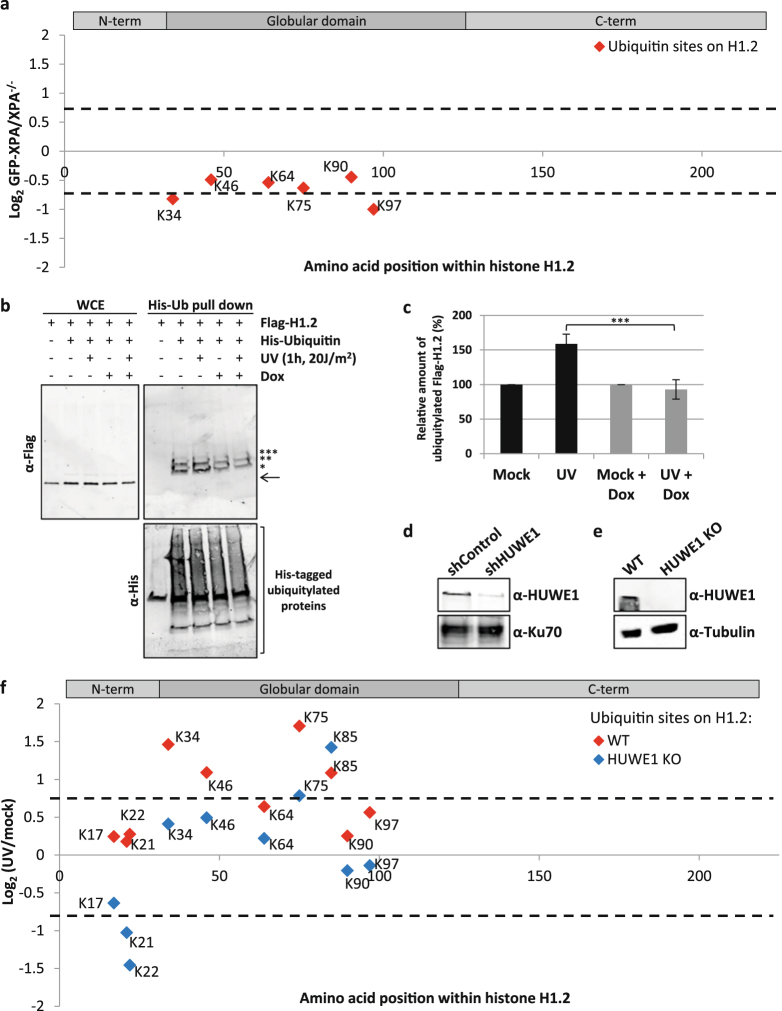
The UV-dependent histone H1 ubiquitylation is dependent on the E3-ligase HUWE1. **(a)** Graphical representation of log_2_ GFP-XPA/XPA^−/−^ SILAC ratio of quantified ubiquitylation sites plotted against the respective lysine positions within histone H1.2. Histone H1 domains are plotted above. Quantification by Maxquant analysis from 1 experiment. **(b)** U2OS cells were transfected with His-Ub and FLAG-H1.2 were UV-C irradiated (20 J/m^2^). To induce expression of the shRNA targeting HUWE1, cells were cultured with doxycycline (1 μg/ml) for 3 days prior to lysis. His-tagged ubiquitylated proteins were isolated 1 hour after UV exposure and analysed by immunoblotting using anti-His and anti-FLAG antibodies. The arrow (←) indicates the unmodified form of FLAG-H1.2. The asterisks (*) indicate modified forms of FLAG H1.2 and the number of asterisks indicate the expected number of conjugated ubiquitin molecules. Cropped westernblots are shown, full blots can be found in supplemental information. **(c)** Quantification of ubiquitylated FLAG-H1.2 signals on western blot after His-Ubiquitin enrichment. Data was normalized to the mock treated signals. The average intensity of 8 independent experiments is plotted and error bars represent standard error of the mean. A two-tailed t-test was used (P = 0.00498) **(d)** Western blot showing the knock down efficiency of the shRNA targeting HUWE1. The shHUWE1 is expressed by culturing cells in doxycycline (1 μg/ml) for 3 days. A sample from cells expressing a non-targeting shRNA is taken along as a control. Ku70 staining is used as a loading control. **(e)** Western blot made from whole cell lysates from WT and HUWE1 KO HeLa cells. Blot was stained with antibodies against HUWE1 and Tubulin. Cropped westernblots are shown, full blots can be found in supplemental information. **(f)** Graphical representation of log_2_ UV/mock SILAC ratio of quantified ubiquitylation sites within histone H1.2 in WT (red) or HUWE1 KO (blue) cells. Quantification by Maxquant analysis from 2 independent experiments. Histone H1 domains are plotted above.

Several UV-induced histone modifications are activated by ssDNA gaps mediated by the NER dependent excision of DNA damage, including H2A ubiquitylation^2,10,40^ and H2AX phosphorylation^9^. To test whether the UV-induced H1 ubiquitylation also depends on NER, we performed di-Gly proteomics experiments and compared XPA-deficient cells (XP-A), in which excision of DNA damage is absent, to XP-A cells rescued by stable GFP-XPA expression^42^. Quantitative mass spectrometry showed that UV-induced H1 ubiquitylation is similar in XPA-deficient and proficient cells, indicating that the H1 ubiquitylation is NER-independent. Some lysines are even slightly more ubiquitylated in XPA deficient cells (Fig. 2a and Supplemental Table S2).

Recently, it was shown that H1 ubiquitylation following double strand break induction was dependent on the E3 ligase RNF8^32^. Also after UV-induced DNA damage RNF8 is activated^10^, however this activation is dependent on NER. This makes RNF8 a less likely candidate to be responsible for our observed H1 ubiquitylation. Another E3-ligase, previously shown to be able to ubiquitylate histone H1 *in vitro*, is HUWE1 (HECT, UBA, and WWE domain containing protein 1)^43^. *In vivo*, HUWE1 was found to be involved in the ubiquitylation of histone H2AX in both unperturbed conditions and upon replication stress^44,45^. In addition, HUWE1 regulates the DDR by targeting different proteins involved in cell cycle checkpoint control, homologous recombination and base excision repair, such as Cdc6^46^, BRCA1^47^, TopBP1^48^ and POLB^49^. To investigate whether HUWE1 is responsible for the UV-induced ubiquitylation of histone H1 we tested the ubiquitylation status of FLAG-H1.2 in cells expressing a doxycycline inducible shRNA targeting HUWE1^50^ (Fig. 2d). Interestingly, HUWE1 knockdown resulted in an almost complete absence of the UV-induced Flag-H1.2 ubiquitylation (Fig. 2b,c). To exclude doxycycline induced effects on H1 ubiquitylation we performed a similar experiment using shControl and shHUWE1 cells, both treated with doxycycline (Supplemental Fig. S2). Also in this experiment the UV-induced H1 ubiquitylation is absent following HUWE1 depletion. Furthermore, the observed UV-independent decrease of H1 ubiquitylation, suggests a possible role for HUWE1 in the constitutive H1 ubiquitylation as well. To further confirm the HUWE1-dependency on ubiquitylation of endogenous H1 species we used quantitative di-Gly proteomics. UV/mock SILAC ratios of the vast majority of ubiquitylated histone H1 peptides were strongly reduced in HUWE1 KO cells as compared to WT HeLa cells (Fig. 2e,f). Of all identified UV-induced ubiquitylation sites, only lysine 85 was not influenced by HUWE1 knock out (KO) (Fig. 2f and Supplemental Table S3), suggesting that probably an additional E3 ligase is involved in H1 ubiquitylation. However, HUWE1 KO severely reduced the levels of histone H1 ubiquitylation on 9 different lysines, indicating that the ubiquitylation levels of histone H1 after UV damage is mainly regulated by HUWE1.

### HUWE1 stimulates the RNF8-RNF168 signalling cascade

Although histone H1 was thus far not found to be implicated in the UV-DDR, it was recently described to be modified with K63-linked poly-ubiquitin chains following DSB induction^32^. The E3-ligase RNF8 is recruited to DSBs in a MDC1 and yH2AX-dependent fashion where it, together with the E2-conjugating enzyme UBC13, poly-ubiquitylates histone H1^32^. The resulting K63-linked poly-ubiquitylated H1 is subsequently bound by the ubiquitin binding domain (UDM1) of the E3-ligase RNF168 which in turn ubiquitylates H2A at lysines K13/K15, stimulating recruitment of downstream factors, such as 53BP1^32,41,51^. Interestingly, using quantitative di-Gly proteomics, it was previously shown that UBC13 depletion barely affects the ubiquitin entities directly coupled to lysines of target proteins^32^. Based on this finding it was hypothesized that UBC13 together with RNF8 mainly conjugates K63-linked ubiquitin chains on pre-existing histone H1 ubiquitin entities. As our ubiquitylation assay suggests that in response to UV damage, HUWE1 mainly stimulates histone H1 modification with either mono-ubiquitin or short poly-ubiquitin chains (Fig. 2b), it is possible that HUWE1 provides the initial ubiquitin modification. These ubiquitylated or ‘primed’ histone H1 molecules would then form a substrate for the subsequent poly-ubiquitylation by RNF8 resulting in the accumulation of downstream factors like 53BP1.

**Figure 3:**
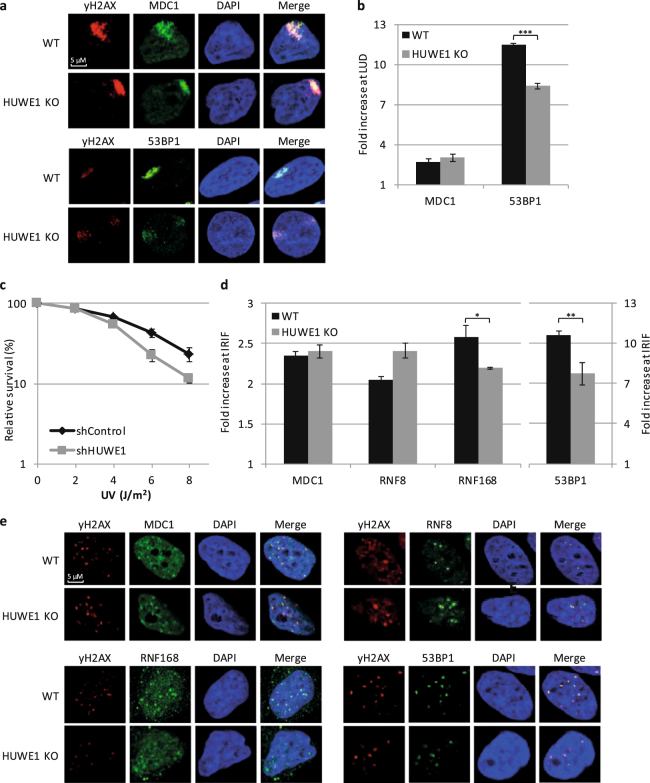
HUWE1 mediated histone H1 ubiquitylation stimulates 53BP1 accumulation to sites of DNA damage. (**a**) Representative images of immunofluorescence experiments to study colocalisation of MDC1 (upper panel) or 53BP1 (lower panel) with yH2AX in WT or HUWE1 KO cells. yH2AX is used as damage marker. Local UV damage was induced (60 J/m^2^) through a 5 µM micropore filter. Cells were incubated with EdU (20 μM) and fixed 2 h after irradiation. **(b)** Quantification of fold increase of MDC1 and 53BP1 at sites of local UV damage (LUD) in non-S-phase cells. Sites of DNA damage are defined by yH2AX signal. The fold increase is calculated as the ratio of the fluorescent intensity at site of damage over the fluorescent intensity in the rest of the nucleus. Average of 3 independent experiments, with at least 25 cells analysed per experiment, is plotted. Error bars represent SEM. P-value(0.0001) was calculated using a two-tailed t-test. **(c)** Clonogenic UV-survival experiments in U2OS cells expressing either shControl or shHUWE1. The percentage of surviving colonies is plotted against the UV-C dose. The number of colonies counted at 0 J/m^2^ is set as 100% survival. Data represents the average of three independent experiments all done in triplicate and error bars represent standard error of the mean. **(d)** Quantification of fold increase of MDC1, RNF8, RNF168 and 53BP1 at sites of ionizing radiation induced foci (IRIF). Double strand breaks are induced by 1 Gy of ionizing radiation and cells were fixed 30 min after damage induction. Sites of DNA damage are defined by yH2AX signal. The fold increase is calculated as the ratio of the fluorescent intensity at sites of damage over the fluorescent intensity in the rest of the nucleus. Average of 3 independent experiments, with at least 75 cells analysed, is plotted. Error bars represent SEM. A two-tailed t-test was used to determine significance of the difference (*P = 0.058 and **P = 0.018. **(e)** Representative images of immunofluorescence experiments to study colocalisation of MDC1, RNF8, RNF168 and 53BP1 with yH2AX 30 min after 1 Gy in WT or HUWE1 KO cells.

Since RNF8-mediated signalling, including H2A ubiquitylation and 53BP1 recruitment, was observed in the UV-DDR^10^, it is not unlikely that histone H1 is also K63-linked ubiquitylated by RNF8 after UV damage and that HUWE1 might be involved in priming H1, thereby stimulating this pathway. In line with this hypothesis, immunofluorescence experiments showed reduced levels of 53BP1 at sites of local UV damage (LUD) in HUWE1 KO cells as compared to control cells, while the amount of MDC1, a factor upstream of H1 ubiquitylation, was not affected at LUD (Fig. 3a,b). To focus on NER-induced signalling, we used Edu labelling to identify S-phase cells, which were excluded in the analysis to eliminate replication stress generated 53BP1 accumulation at UV-damaged DNA (Supplemental Fig. S3a). Although the intensity of 53BP1 at damaged sites is clearly reduced in HUWE1 KO cells, it is important to note that the colocalization is not lost. This indicates that the DNA damage signalling is not completely absent, as was observed upon knockdown of RNF8^10^, but rather suggests that the signal amplification is affected. Similar results were found in U2OS cells that express a doxycycline inducible shRNA targeting HUWE1 (Supplemental Fig. S3b,c). RNF8 depletion has previously been reported to result in an increased sensitivity to DNA damaging agents^10,52–54^ and therefore we also tested the UV-sensitivity upon HUWE1 depletion using colony survival assays. Both HUWE1 knockdown by dox inducible shRNA expression (Fig. 3c) or transient siRNA transfection (Supplemental Fig. S3d,e), each targeting different sequences to exclude off target effects of the siRNA and shRNA, show a mild sensitivity to UV damage compared to control cells.

The RNF8-RFN168-dependent signalling pathway is best described in response to DSBs. To test whether HUWE1 is also involved in the cellular responses to DSBs, we studied the recruitment of the proteins involved in the RNF8-RNF168 pathway to ionizing radiation-induced foci (IRIF) in the presence or absence of HUWE1. Staining for 53BP1 and RNF168, DDR factors that are recruited to DSBs in a histone H1 K63-linked ubiquitylation dependent manner^32^, showed reduced signals at IRIF in HUWE1 KO cells compared to control cells (Fig. 3d,e). As expected, factors that are recruited upstream of histone H1 poly-ubiquitylation, like MDC1 and RNF8, did not show this reduced recruitment. In line with the effects of HUWE1 during UV-induced signalling, the relative amount of accumulated 53BP1 and RNF168 at IRIF was decreased. However, the number of IRIF showing colocalisation of 53BP1 and RNF168 with yH2AX did not change. Together our data shows that HUWE1 stimulates histone H1 ubiquitylation and affects the recruitment levels of factors downstream of the RNF8-mediated H1 poly-ubiquitylation in response to both UV and IR-induced DNA damage.

## Discussion

Our di-Gly quantitative proteomics approach identified H1 as one of the most prominent ubiquitylated proteins following UV-induced DNA damage. This DNA damage-dependent H1 ubiquitylation was found at multiple lysines on histone H1 (Fig. 1b,c). H1 ubiquitylation was confirmed by our His-Ub pull downs (Fig. 1d) that identified H1 species modified with 1, 2 and 3 ubiquitin entities. Together, with the fact that HUWE1 depletion resulted in a decrease of the UV-induced H1 ubiquitylation (Fig. 2), this might suggest that HUWE1 mono-ubiquitylates histone H1 on 1 to 3 different lysine residues, however we cannot exclude that HUWE1 also generates poly-ubiquitin chains on H1. The slower migrating histone H1 bands that were observed in Figs 1 d and 2a probably do not represent RNF8-mediated K63 poly-ubiquitylation, as these poly-ubiquitin chains display higher molecular weight bands^32^. Therefore, HUWE1-mediated ubiquitylated forms of histone H1 are probably not directly bound by RNF168, as the UDM1-domain of RNF168 preferentially binds to longer ubiquitin chains^32,55^. Based on our data we propose a model in which HUWE1 is important for the initial damage-induced ubiquitylation of histone H1. This H1 ‘priming’ by ubiquitylation may provide additional substrates for more efficient K63-linked poly-ubiquitylation by UBC13/RNF8, as these proteins most likely elongate pre-existing ubiquitin entities to a K63-linked ubiquitin chain^32,56–58^. Since HUWE1 is involved in the UV-induced ubiquitylation of multiple lysines on histone H1, it is also likely that chain-extension by RNF8 is not site-specific and might generate ubiquitin chains on several histone H1 sites.

**Figure 4:**
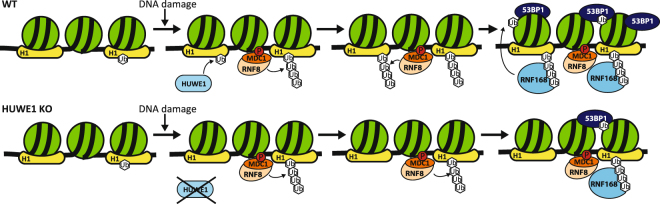
UV-induced H1 ubiquitylation is mediated by HUWE1 and stimulates the RNF8-RNF168 pathway. Model of HUWE1 functioning in the DNA damage-induced ubiquitin pathway. HUWE1 ubiquitylates histone H1, with a mono-ubiquitin or short poly-ubiquitin chain, in response to DNA damage, thereby providing additional substrates for RNF8-mediated K63-linked poly-ubiquitylation. These K63-chains are recognized and bound by RNF168 which ubiquitylates histone H2A at lysine K13/15, stimulating the recruitment of downstream factors like 53BP1. P = phosphorylation of H2AX, Ub = ubiquitylation.

It is not yet known how the E3 ligase activity of HUWE1 towards histone H1 is regulated. Interestingly, UV-induced H1 ubiquitylation is not dependent on active NER (Fig. 2a). This is in contrast to the activation of the RNF8-RNF168-mediated signalling following UV-induced DNA damage, which is dependent on the presence ssDNA gaps generated by the excision of damaged DNA by the XPF/ERCC1 and XPG endonucleases^2,9,10,40^. This difference in NER-dependency of the HUWE1 and RNF8-mediated H1 ubiquitylation suggests that HUWE1 is presumably activated by a different mechanism than RNF8. Moreover, as our UV-induced H1 ubiquitylation events detected in our di-Gly proteomics approach are NER-independent, these most likely do not represent H1 ubiquitylation events generated by RNF8. Our data further suggest that HUWE1 is also involved in the constitutive ubiquitylation of H1 (Supplemental Fig. S2). The increased H1 ubiquitylation in response to DNA damage (Fig. 2b–f) may be derived by activation of HUWE1, which may also explain the previously noted targeting of other DDR proteins by HUWE1 following DNA damage^46–49^. However, the HUWE1-mediated H1 ubiquitylation following DNA damage might also be explained by enhanced accessibility of the targeted lysines of histone H1 due to chromatin changes after DNA damage induction. Of note, recruitment of factors downstream of RNF8 to sites of DNA damage was still observed in the absence of HUWE1, in contrast to RNF8-depleted cells in which recruitment of these factors is almost completely absent^10,52–54^. However, the amount of accumulation of downstream factors, such as 53BP1, is significantly reduced (Fig. 3b–e). While depletion of HUWE1 severely inhibits the DNA-damage induced histone H1 ubiquitylation, ubiquitylated histone H1 molecules are not completely lost (Fig. 2b). These residual HUWE1-independent H1 ubiquitylation may still be sufficient to serve as a substrate for RNF8 and Ubc13. This indicates that HUWE1 can be considered as an important but non-essential player in the RNF8-RNF168 pathway, in which it stimulates signal amplification. Since HUWE1 targets multiple proteins in the DDR, we cannot rule out that these targets might also contribute to the signal amplification. In summary, our data are in line with a two-step model of histone H1 ubiquitylation in response to DNA damage, in which HUWE1 primes histone H1 to stimulate RNF8-Ubc13-mediated K63-linked ubiquitylation and adds an extra player to the ubiquitin-regulated DNA damage induced signalling pathway^59,60^ (Fig. 4).

## Methods

### Cell culture

All cells were cultured in DMEM/F10 supplemented with 10% fetal calf serum (FCS) and 1% penicillin-streptomycin (PS, P0781 Sigma) at 37 °C and 5% CO_2_ in a humidified incubator. XP2OS (sv40) cells and the functionally complemented GFP-XPA expressing XP20S cells were described earlier^42^. For SILAC labelling, cells were cultured in DMEM deficient in lysine, arginine and L-glutamine (PAA), supplemented with 10% dialyzed fetal calf serum (Invitrogen), PS and ultra-glutamine (Lonza). Cells were grown in medium containing either 73 µg/ml light [^12^C6]-lysine and 42 µg/ml [^12^C6, ^14^N4]-arginine (Sigma) or similar concentrations of heavy [^13^C6]-lysine or [^13^C6, ^15^N_2_]-lysine and [^13^C6, ^15^N4]-arginine (Cambridge Isotope Laboratories) for at least 10 cell doublings. HUWE1 KO HeLa cells were a kind gift of xthe Moldovan lab^45^ and cultured in 3% oxygen incubators. The U2OS cells expressing shRNA targeting HUWE1 were a kind gift from the lab of Xiaodong Wang and cultured with doxycycline (1 μg/ml) for 3–5 days prior to UV irradiations^50^. SiRNA transfections were performed with RNAiMax (Invitrogen) 3 days prior to UV treatments according to the manufacturer’s protocol. The sequences of the used siRNAs were: siControl UGGUUUACAUGUCGACUAA and siHUWE1 GAGUUUGGAGUUUGUGAAGTT^46^.

### Isolation of ubiquitylated peptides

For di-Gly enrichments either U2OS (Fig. 1a–c and Supplemental Table S1), XP2OS (Fig. 2a and Supplemental Table S2) or HeLa (Fig. 2f and Supplemental Table S3) cells were used. One hour prior to harvesting, SILAC labelled cells were washed with PBS and UV-irradiated (16 J/m^2^, 254 nm, Philips TUV lamp) or mock treated. Cells were harvested by trypsinisation, resuspended in culture medium and cell number was determined using a Z2 coulter particle counter and size analyzer (Beckman coulter). Cells were washed twice with cold phosphate-buffered saline (PBS), and heavy and light labelled cells were mixed in a 1:1 ratio and stored at −80 °C until use. Cells were lysed in 7.5 ml denaturing buffer, containing 8 M UREA, 50 mM Tris pH[8.0], 50 mM NaCl, 50 µM MG132 (Biomol), 20 μM PR-619 (LifeSensors) and complete protease inhibitor cocktail (Roche), for 10 min on ice. Following lysis, samples were sonicated 3x for 30 sec with a Soniprep 150 (MSE) and centrifuged at 2500 g at 4 °C for 10 min to remove insoluble material. 15–20 mg of protein, as determined by a bicinchoninic acid (BCA) protein assay (Pierce), was reduced with 5 mM dithiothreitol (DTT) and alkylated with 5.5 mM chloroacetamide. Cell lysates were diluted to 4 M urea with 50 mM Tris pH[8.0] and digested with endoproteinase Lys-C (10 μg/mg protein, Wako Chemicals) for 1 hour at RT. Samples were further diluted to 1.6 M urea and incubated overnight at 30 °C with proteomics grade trypsin (Roche) at an enzyme to substrate ratio of 1:100. Protease digestion was stopped by addition of trifluoracetic acid (TFA) to a final concentration of 1%. Peptides were purified with 500 mg tC18 SEP-PAK SPE cartridges (Waters) and eluted with 40% acetonitrile (ACN) containing 0.1% TFA. Subsequently, peptides were lyophilized for 48 hours (Scanvac CoolSafe 110-4, Scala Scientific). Lyophilized peptides were dissolved in 1.4 ml of IAP buffer (PTMscan, cell signalling) and incubated with anti-K-ε-GG antibody beads (PTMscan, cell signalling) for 2 hours at 4 °C on a rotating unit. Beads were washed three times in IAP buffer followed by two washes in H_2_O and immunoprecipitated peptides were eluted using 0.1% of trifluoracetic acid (TFA) in H_2_O. Eluted peptides were purified using C18 stagetips (ziptips_C18_).

### Mass spectrometry

Samples were analysed with a Orbitrap Lumos Tribid mass spectrometer (Thermo Fisher Scientific) or a quadrupole Orbitrap (Q-Exactive, Thermo Fisher Scientific) according to protocols below.

Mass spectra were acquired on an Orbitrap Lumos Tribid mass spectrometer (Thermo Fisher Scientific) coupled to an EASY-nLC 1200 system (Thermo Fisher Scientific). Peptides were separated on an in-house packed 75 μm inner diameter column containing 50 cm CSH130 resin (3.5 μm, 130 Å, Waters) with a gradient of 2−20% (ACN, 0.1% FA) over 150 min at 300 nL/min. The column was kept at 50 °C in an NanoLC oven - MPI design (MS Wil GmbH). For all experiments, the instrument was operated in the data-dependent acquisition (DDA) mode. MS1 spectra were collected at a resolution of 120,000, with an automated gain control (AGC) target of 2E5 and a max injection time of 50 ms. The most intense ions were selected for MS/MS, top speed method 3 seconds cycle time. Precursors were filtered according to charge state (2–7z), and monoisotopic peak assignment. Previously interrogated precursors were dynamically excluded for 70 s. Peptide precursors were isolated with a quadrupole mass filter set to a width of 0.7 Th.

Peptide samples were analysed on a quadrupole Orbitrap (Q-Exactive, Thermo Fisher Scientific) mass spectrometer equipped with an EASY-nLC 1000 (Thermo Fisher Scientific). Peptide samples were loaded onto ReproSil C18 reversed phase column (20 cm × 75 μm) and eluted with a linear gradient (3 h) from 5 to 80% acetonitrile containing 0.1% formic acid at a constant flow rate of 300 nl/min. Fragmentation of the peptides was performed in a data-dependent acquisition (DDA) mode. MS1 spectra were collected at a resolution of 70,000, with an automated gain control (AGC) target of 1E6 and a max injection time of 50 ms. The 10 most intense ions were selected for MS/MS. Precursors were filtered according to charge state (2–7z), and monoisotopic peak assignment. Previously interrogated precursors were dynamically excluded for 30 s. Peptide precursors were isolated with a quadrupole mass filter set to a width of 2.0 Th.

### Data analysis

Raw data files were analysed using MaxQuant software (version 1.5.1.0)^33^. MS/MS spectra were searched against the human International protein Index (IPI) database (version 3.68), using Andromeda search engine^61^. Spectra were searched with a mass tolerance of 6 ppm. The specificity was set to trypsin, and a maximum of 4 missed cleavages was allowed. Cysteine carbamidomethylation was set as a fixed modification whereas methionine oxidation, N-terminal protein acetylation and di-glycine-lysine were set as variable modifications in Maxquant analysis. A false discovery rate of 0.05 for peptides and a minimum peptide length of 6 were set. Before data analysis, known contaminants and reverse hits were removed from the modification specific peptide list. Scatter plots of the ubiquitylated peptides were generated using Perseus software (version 1.5.4.1). Ubiquitylation sites that were upregulated in response to UV were subjected to Gene Ontology (GO) enrichment analysis (GO_BP4) using the functional annotation tool of DAVID bioinformatics resources^62^. Enriched terms were sorted by p-value.

### Isolation of hexa-His-tagged proteins

U2OS cells (70% confluent, 10 cm dish) were transfected with 6xHis-tagged ubiquitin (15 μg) and FLAG-tagged histone H1.2 (5 μg) constructs using X-treme gene HP (Roche), one day before cell lysis according to manufacturer’s protocol. One hour before lysis, the cells were treated with 20 J/m^2^ UV. Cells were washed in PBS and harvested by scraping in 750 µl denaturing urea buffer (8 M urea, 300 mM NaCl, 50 mM Na_2_HPO_4_, 0.5% NP-40; pH 8.0) supplemented with 10 µM MG132 (Biomol), 10 mM N-ethylmaleimide (Sigma) and complete protease inhibitor cocktail without EDTA (Roche). Lysates were sonicated 3 times for 10 sec with amplitude 12 and centrifuged at 13,000 g and 4 °C for 15 min to remove remaining cell debris. Meanwhile 50 µl of Co^2+^ Sepharose beads slurry was equilibrated 3 times with urea buffer. Cleared lysates were incubated with Co^2+^ Sepharose beads for 2 h at 4 °C. Subsequently, beads were washed 4 times with urea buffer for 5 min and centrifuged at 3000 rpm for 1 min. His-tagged proteins were eluted by 20 min incubation with urea buffer containing 500 mM EDTA. Eluents were mixed with Laemmli buffer and separated on a Precast BioRad gel 5–14% and transferred to a PVDF membrane (0.45 µm).

### Immunoblotting

Cells were lysed in Leammli buffer, separated on 6% SDS-Page gels and transferred to a PVDF membrane. Membranes were blocked with 5% milk in PBS at RT for 1 h and incubated with primary antibodies for 1–2 h. Primary antibodies used: mouse-anti-6xHis-tag (Qiagen #34660, 1:1000), rabbit-anti-FLAG (Sigma E1804, 1:1000), rabbit-anti-HUWE1 (Bethyl A300-486A-2, 1:1000), rabbit-anti-XPC^63^ and goat-anti-Ku70 (Santacruz sc-1487, 1:1000). Alexa Fluor 795 donkey anti-mouse antibodies and Alexa Fluor 680 donkey anti-rabbit (LI-COR Biosciences) were used to visualize the proteins using an infrared imaging system (Odyssey; LI-COR Biosciences).

### Immunofluorescence

Cells were grown on coverslips until 70–80% confluency. After PBS washing, local UV damage was inflicted by irradiation through 5 µM micropore filters (Milipore). Directly after UV irradiation cells were incubated with EdU (20 μM) containing medium to visualize cells in S-phase. After 2 h the cells were washed in PBS and fixed in 2% paraformaldehyde in PBS containing 0.1% triton-X and permeabilized for 20 min in 0,5% Triton-X in PBS. EdU was visualized with a click-it reaction (Click-it EdU imaging kit, Invitrogen) using a 647 nm fluorescent azide (Biotium) according to manufacturer’s protocol. After the EdU labelling procedure, cells were washed in PBS containing 0.5% bovine serum albumin (BSA) and 0.15% glycine and stained with primary antibodies for 2 h at RT. Coverslips were washed three times short and twice for 10 min in 0.1% triton-X in PBS and once in PBS with BSA and glycine and subsequently stained with secondary antibodies labelled with alexa fluorochromes 488 and 555 (Invitrogen) and DAPI (0.1 μg/ml) for 1 h at RT. Coverslips were mounted with Aqua Poly/Mount (Polysciences). In experiments using ionizing radiation, cells were fixed 30 min after 1 Gy in 2% paraformaldehyde in PBS containing 0.1% triton-X and permeabilized in 0,1% Triton-X in PBS. Images were obtained using a LSM700 microscope (Carl Zeiss Microimaging Inc.) equipped with a 63 × oil immersion lens (Plan-apochromat, 1.4 NA) and analysed using ImageJ software^64^. In short data was analysed using a macro that first defined the cell nucleus by DAPI signal, then identified damage sites (local UV damage or IR induced foci) by yH2AX signal and finally measured the fluorescent signal of the protein of interest at these sites. EdU positive cells were manually excluded. Antibodies used: mouse-α-yH2AX (1:1000, Millipore 05–636); rabbit-α-53BP1 (1:1000, Santacruz sc-22760); rabbit-α-MDC1 (1:500, Abcam ab11171); rabbit-α-yH2AX (1:1000, Abcam ab11174); mouse-α-RNF8 (1:50, Santacruz sc-271462); mouse-α-RNF168 (1:100, Milipore ABE367).

### Clonogenic survival assays

For each condition 500 cells/well were seeded in 6-well plates in triplicate. Cells were irradiated with different doses of UV-C one day after seeding and cultured for five days. The cells were fixed and stained in 50% methanol, 43% water, 7% acetic acid and 0.1% brilliant blue (Sigma). The colonies were counted using GelCount^TM^ (Oxford Optronix, version 1.1.2.0).

### Statistics

Each experiment was performed at least three times and mean values and standard error of the means (SEM) are shown. To determine if differences between conditions are significant a two-tailed t-test was used. P-values < 0.1 (*), < 0.05 (**) and < 0.005 (***) were considered as significant different.

### Data availability

All data generated or analysed during this study are included in this published article (and its Supplementary Information files). Raw datasets generated are available from the corresponding author on reasonable request.

## Electronic supplementary material

Supplementary figures

Supplemental Table S1

Supplemental Table S3

Supplemental Table S
