# Supplementary material for: DNA damage-induced histone H1 ubiquitylation is mediated by HUWE1 and stimulates the RNF8-RNF168 pathway
Source: Sci Rep. 2017 Nov 10;7:15353. doi: 10.1038/s41598-017-15194-y (PMC5681673; doi:10.1038/s41598-017-15194-y)

DNA damage-induced histone H1 ubiquitylation is mediated by HUWE1 and stimulates the RNF8-RNF168 pathway

I.K. Mandemaker[1], L. van Cuijk[1], R.C Janssens[1], H. Lans[1], K. Bezstarosti[2], J.H. Hoeijmakers[1], J.A. Demmers[2], W. Vermeulen[1], J.A. Marteijn[1,*]

[1]*Department of Molecular Genetics, Cancer Genomics Netherlands, Erasmus Medical Centre, Rotterdam, The Netherlands*

[2]*Department of Proteomics, Erasmus Medical Centre, Rotterdam, The Netherlands*

* To whom correspondence should be addressed: Jurgen A. Marteijn; Tel: +31 107038169; Email: J.Marteijn@erasmusmc.nl

Supplemental figure 1: Isolation of UV-responsive ubiquitylated peptides

(a) Workflow of SILAC based proteomics approach combined with the isolation of di-Gly peptides to identify UV-responsive ubiquitin sites. Mock treated cells were grown in light (K0R0) medium and UV treated (16J/m^2) cells in heavy (K6R10) medium. One hour after treatments cells were mixed in a 1:1 ratio and lysed in denaturing buffer. Proteins were digested with Lys-C and trypsin after which di-Gly immunopurification was performed. The enriched di-Gly peptides were identified and analysed by LC-MS/MS. **(b)** Experimental overlap of the identified di-Gly peptides in three biological replicates (experiment #1 red, #2 green and #3 blue). Venn diagrams show the total number of di-Gly peptides identified (left), UV-induced di-Gly sites (top right) and UV-reduced sites (bottom right) quantified in each experiment. **(c)** Scatter plot with the $\log_2$ SILAC ratio (UV/mock) plotted against the $\log_{10}$ intensity of the identified di-Gly peptides as quantified by Maxquant analysis from 3 independent experiments. Peptides were considered as UV-responsive when its $\log_2$ SILAC ratio was increased or decreased (dotted lines) more than 0.75. Grey: Non-responsive di-Gly peptides; Red: UV-increased ubiquitylated peptides; Blue: UV-reduced ubiquitylated peptides; Black: Increased di-Gly peptides for known UV-dependent ubiquitylated proteins FANCD2, XPC and DDB2. The amino acid position of the modified lysine is shown between brackets. **(d)** Quantification of the identified di-Gly modified ubiquitin peptides originating from poly-ubiquitin chains, which is a measurement for the abundance of the different ubiquitin chain linkages. Values represent the average of three experiments; error bars indicate SD. **(e)** Functional annotation of UV-induced ubiquitylation sites ($\log_2$ SILAC ratio >0.75) into biological pathways using DAVID bioinformatics resource. Enriched GO-terms (BP4) (p-value<0.01) are plotted. Bar graph indicates the fold enrichment of biological processes. The fold enrichment defines the ratio between input genes in a pathway over the whole genome in that pathway. The significance (p-value) of the biological pathways was determined by Fisher's exact test and is indicated in red.

Supplemental figure 2: The UV-dependent histone H1 ubiquitylation is dependent on the E3-ligase HUWE1. U2OS cells, expressing either shControl or shHUWE1, were transfected with His-Ub and FLAG-H1.2 were UV-C irradiated (20 J/m^2). Cells were cultured with doxycycline (1 µg/ml) for 3 days prior to lysis to induce expression of the shRNA. His-tagged ubiquitylated proteins were isolated 1 hour after UV exposure and analysed by immunoblotting using anti-His and anti-FLAG antibodies. The arrow (←) indicates the unmodified form of FLAG-H1.2. The asterisks (*) indicate modified forms of FLAG H1.2 and the number of asterisks indicate the expected number of conjugated ubiquitin molecules based on the shift in mass of histone H1.

Supplemental figure 3: HUWE1 mediated histone H1 ubiquitylation stimulates 53BP1 accumulation to sites of DNA damage. (a) Immunofluorescence images showing DAPI and EdU signal in cells treated with EdU (20 µM) for 2 h. Arrows indicate non-S-phase cells. **(b)** Representative images of immunofluorescence experiments to study colocalisation of MDC1 and 53BP1 with yH2AX in shControl of shHUWE1 expressing cells. Local UV damage was induced (60 J/m^2) through a 5 µM micropore filter. Cells were incubated with EdU (20 µM) and fixed 2h after irradiation.**(c)** Quantification of fold increase of MDC1 and 53BP1 at sites of local UV damage (LUD). Cells that incorporated high levels of EdU, indicative of S-phase cells, were excluded from analysis. Sites of local UV-induced DNA damage are defined by yH2AX signal. The fold increase is calculated as the ratio of the fluorescent intensity at site of damage over the fluorescent intensity in the rest of the nucleus. Average of 3 independent experiments, in which at least 25 cells were analysed, is plotted. Error bars represent SEM. P-value (0.019) was calculated with a two-tailed t-test. **(d)** Clonogenic UV-survival experiments in U2OS cells transfected with either siControl, siHUWE1 or siXPC. The percentage of surviving colonies is plotted against the UV-C dose. The number of colonies counted at 0 J/m^2 is set as 100% survival. The data represent the average of three independent experiments, all done in triplicate, and error bars represent standard error of the mean. **(e)** Western blot showing the knock down efficiency of the siRNAs targeting HUWE1 or XPC. Ku70 is used as a loading control.

Supplemental figure 1

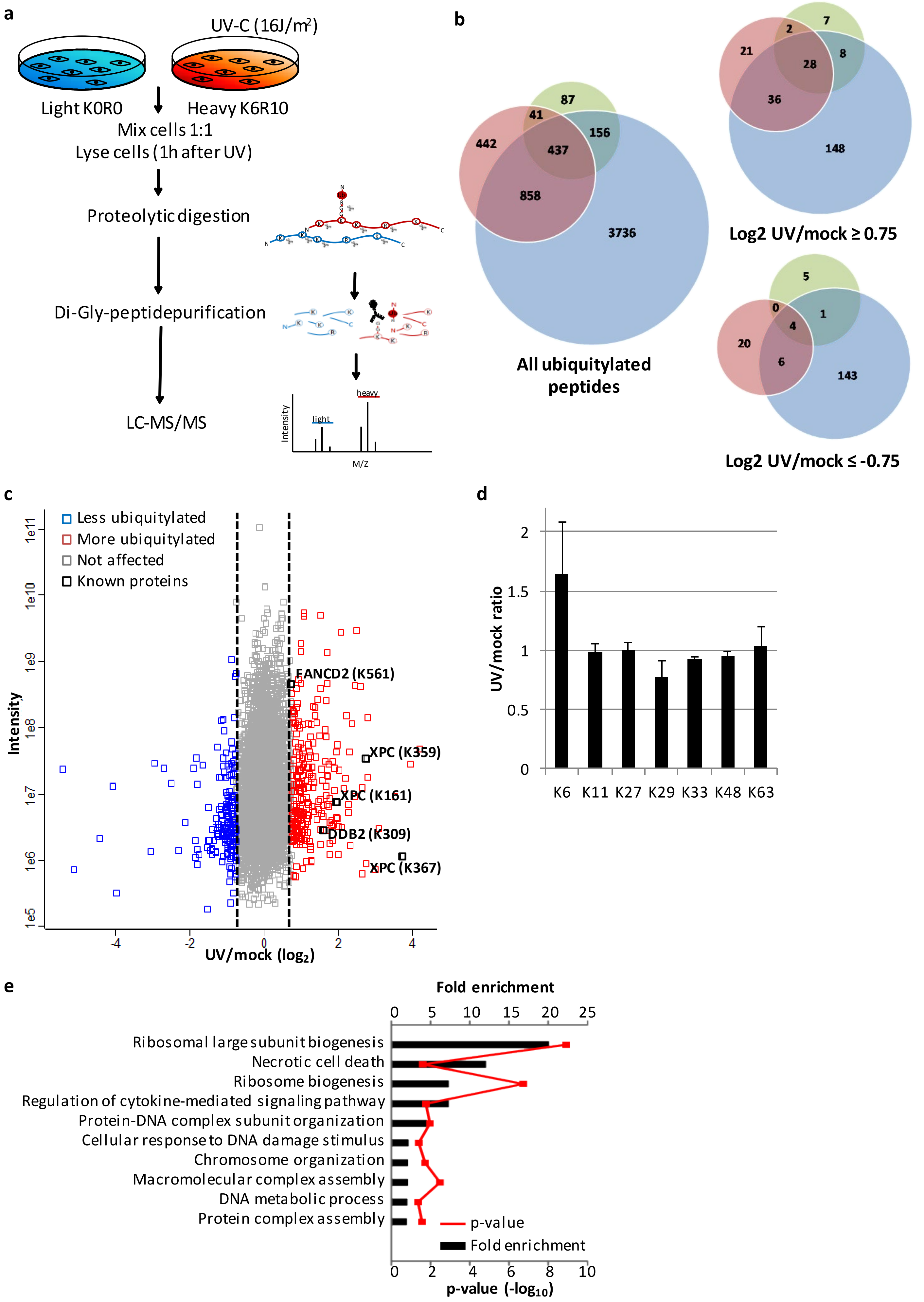

Supplemental figure 2

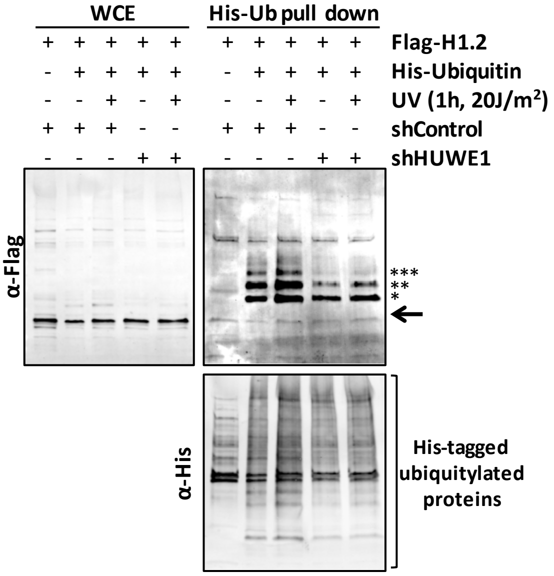

Supplemental figure 3

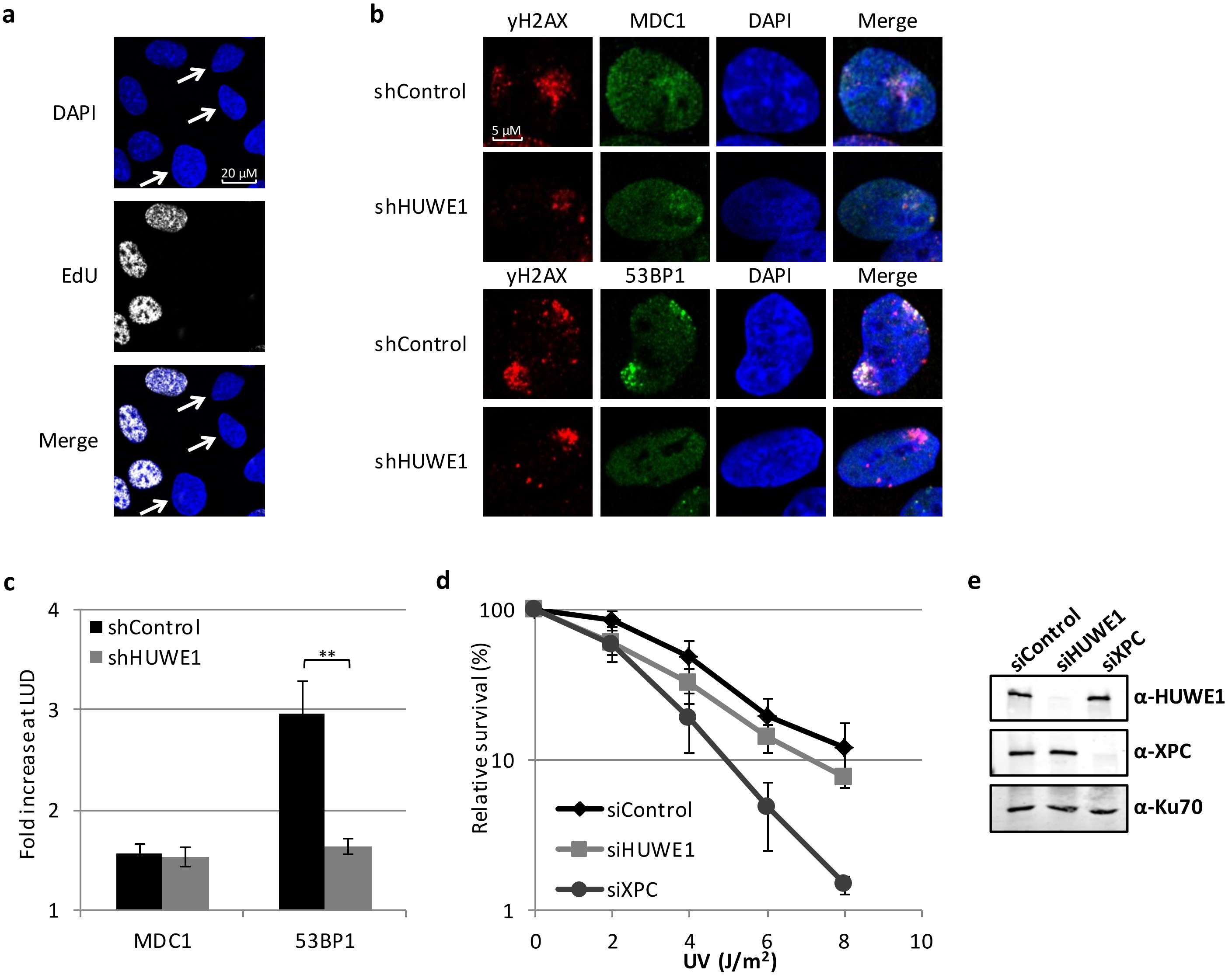

Uncropped westernblots from figure 1d

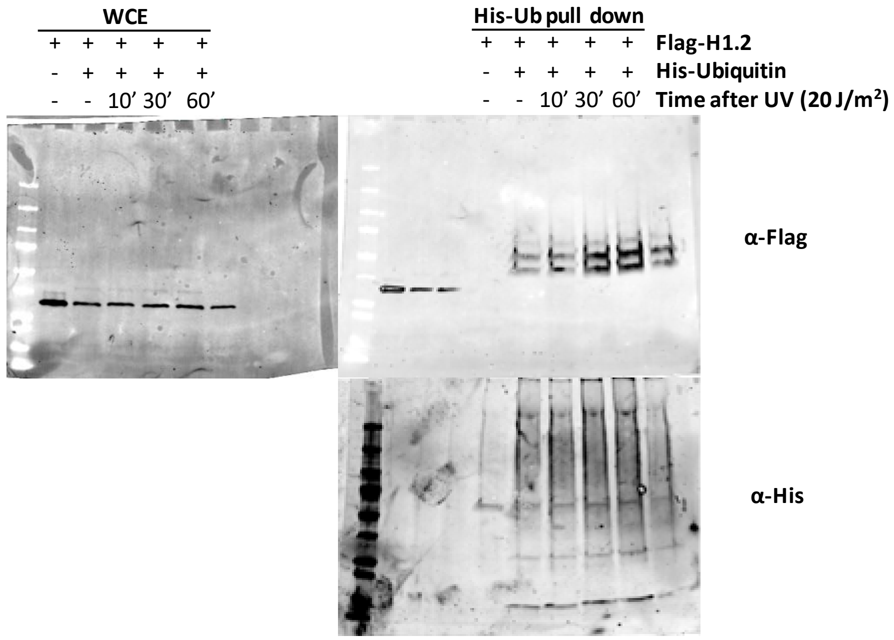

Uncropped westernblots from figure 2b

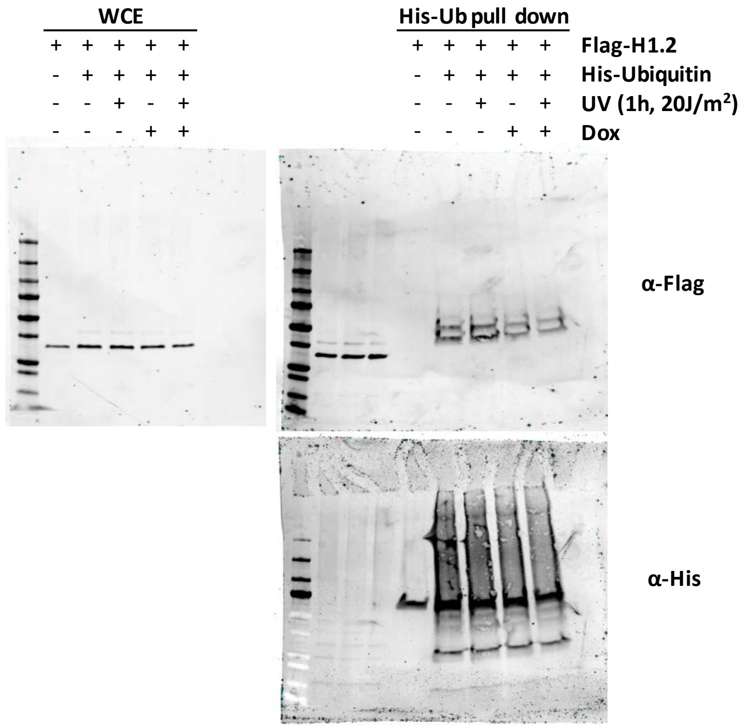

Uncropped westernblots from figure 2d

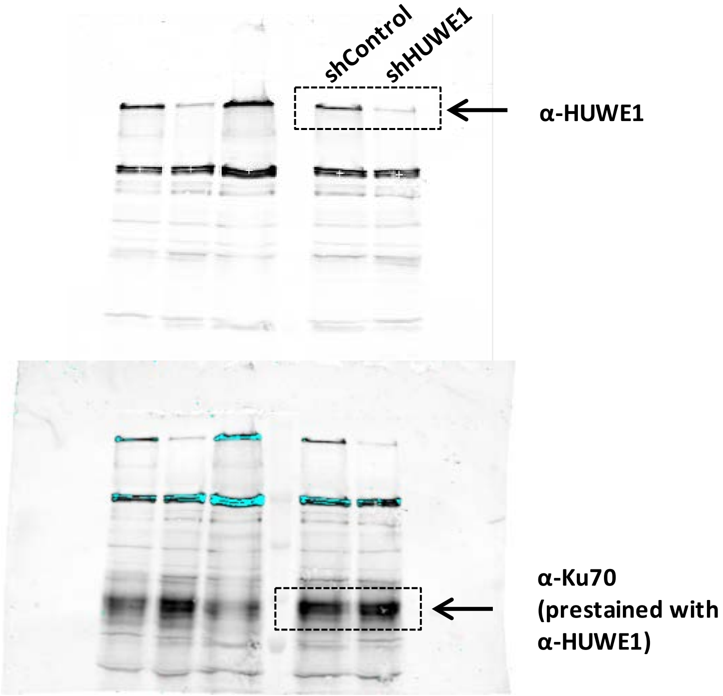

Uncropped westernblots from figure 2e

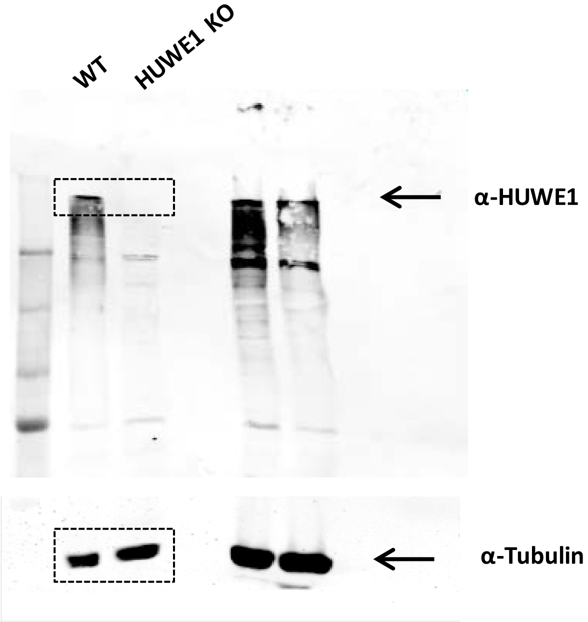

Uncropped westernblots from supplemental figure 2

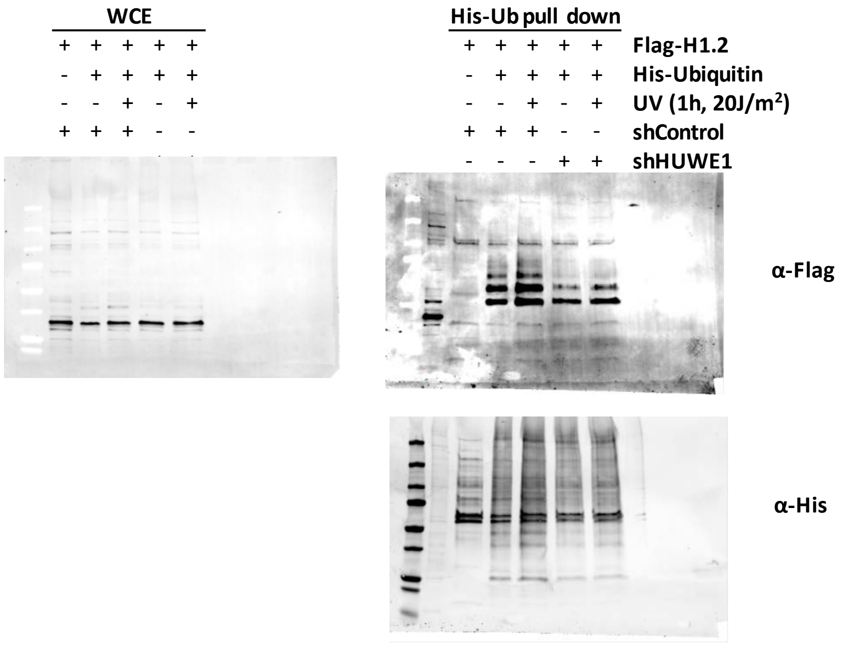

Uncropped westernblots from supplemental figure 3e

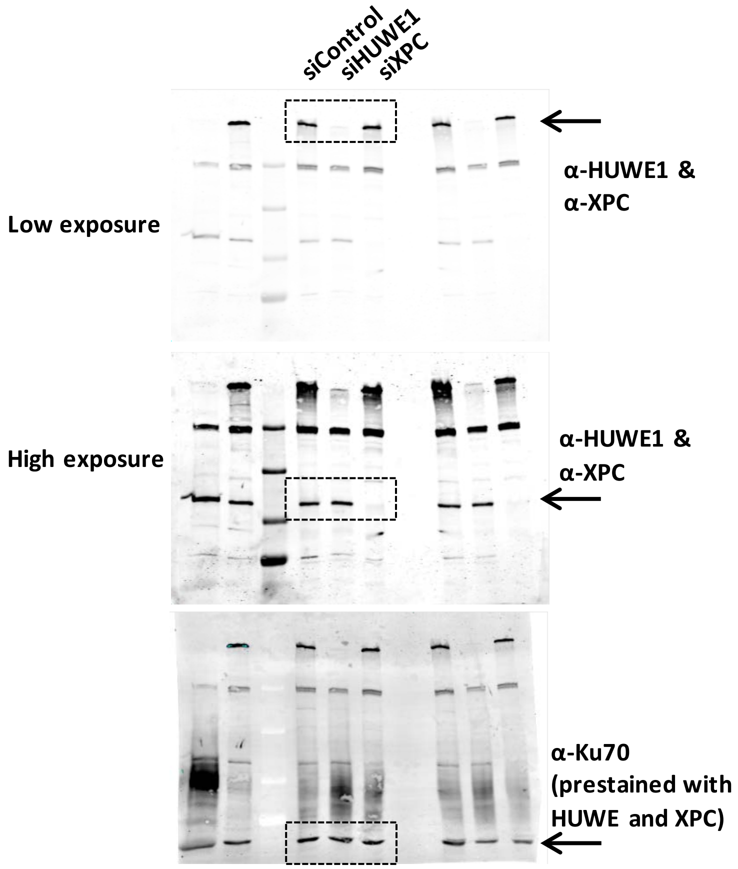

Supplement: Supplementary file 1 — Supplementary figures [file 41598_2017_15194_MOESM1_ESM.pdf]
